# Ten-month follow-up of patients with covid-19 temporally related multi-system inflammatory syndrome in children: the experience of the children hospital of Palermo

**DOI:** 10.1186/s13052-023-01416-9

**Published:** 2023-03-23

**Authors:** Maria Cristina Maggio, Salvatore Giordano, Maria Concetta Failla, Martina Gioacchina Campione, Annalisa Alaimo, Giovanni Corsello

**Affiliations:** 1 University Department PROMISE “G. D’Alessandro”, University of Palermo, Via del Vespro 129, 90100 Palermo, Italy; 2 U.O.C. of Paediatric Infectious Diseases, Paediatric COVID Center, Children Hospital “G. Di Cristina”, ARNAS, Via Dei Benedettini 1, 90100 Palermo, Italy; 3 U.O.C. of Paediatric Cardiac Diseases, Children Hospital “G. Di Cristina”, ARNAS, Via Dei Benedettini 1, 90100 Palermo, Italy

**Keywords:** Multisystem inflammatory syndrome in children (MIS-C), SARS-CoV-2, COVID-19, Methylprednisolone, Kawasaki disease, Intravenous immunoglobulin

## Abstract

**Background:**

In Sicily, the first wave of COVID-19 showed a low epidemic impact in paediatric population, while the second and the third waves had a higher impact on clinical presentation of COVID-19 in children and a significantly higher severe outcome in patients with multisystem inflammatory syndrome in children (MIS-C), with a frequent life-threatening progression.

**Methods:**

We describe a cohort of 22 Sicilian children (11 M; 11 F; age: 1.4–14 years), presenting with clinical features compatible with MIS-C. Patients with negative swab had a history of recent personal or parental infection.

**Results:**

The following diagnostic criteria were detected: fever (100%); cheilitis and/or pharyngeal hyperaemia (86%); latero-cervical lymphadenitis (82%); rash (73%); abdominal pain and/or vomiting and/or diarrhoea (64%); conjunctivitis (64%); hands and feet oedema (18%). 59% showed cardiac involvement (6 pericardial effusion; 8 mitral valve insufficiency; 4 insufficiency of two valves; 3 coronary artery lesions (CAL)). In all the patients, treatment was started within 72 h after the admission, with intravenous immunoglobulins (IVIG) (2 g/Kg/dose), methylprednisolone (2 mg/Kg/day in 73% of patients; 30 mg/Kg/day for 3 days, followed by 2 mg/Kg/day in 27% of patients). Two patients were treated with enoxaparin. Two patients with shock, were additionally treated with vasoactive drugs, albumin, diuretics. Cardiac involvement evolved into the complete resolution of lesions in most of the patients. All the patients were included in a follow-up, to investigate on clinical outcome and resolution of organ involvement. Cardiac valve insufficiency persisted only in 18% of children, CAL persisted only in 33% of children with coronary involvement, however without the evolution into aneurisms.

**Conclusions:**

The preferred treatment strategy was more aggressive at the diagnosis of MIS-C, to block the cytokine cascade. Most of our patients, in fact, received a first-line treatment with IVIG and steroids. This approach could explain the favourable prognosis, the rapid restoring of cardiac function also in patients with MAS or shock, and the good outcome during the 10 months follow-up in all the patients.

## Background

The severe acute respiratory syndrome coronavirus 2 (SARS-CoV-2) induced a rapid pandemic evolution, with infected individuals of all ages in almost all the countries around the world. In Sicily, the first wave of COVID-19 showed a low epidemic impact in our paediatric population, while the second and the third waves had a higher impact on clinical presentation of COVID-19 in children and a significantly higher severe outcome in patients with MIS-C, frequently showing a life-threatening progression.

However, the lower incidence of paediatric cases of severe COVID-19 was and is yet associated with the emergence of the multisystem inflammatory syndrome affecting children in relation to COVID-19 called “multisystem inflammatory syndrome in children” (MIS-C). MIS-C is characterized by hyperinflammation, secondary to cytokine storm and shows some similarities with severe COVID-19 in adult patients [1, 2].

Most children affected were RT-PCR negative for SARS-CoV-2 virus, but were antibody positive, indicating past infection. The pathogenesis of the clinical syndrome was postulated to be a late inflammatory response following SARS-CoV-2 infection.

The main clinical symptoms are persistent fever, conjunctivitis, oral mucosal changes, vomiting, diarrhoea, abdominal pain, respiratory symptoms, myocardial dysfunction, capillary leak and cardiogenic shock. Patients with MIS-C show a significant overlap of symptoms with hyper-inflammatory diseases such as Kawasaki disease (KD), KD-related shock syndrome or Toxic Shock Syndrome (TSS), associated with hypotension, platelet consumption and increased risk of CAL, and Macrophage Activation Syndrome (MAS) [3]. This overlap may occur despite the different aetiology of these conditions, joint by the activation and the dysregulation of pathways implicated in innate immune response, inflammation, and tissue damage induction. Inflammatory markers show significant increase in MIS-C, but none is specific and sensitive for the diagnosis. Treatment approaches chosen in other hyper-inflammatory syndromes have been used to control the hyper-inflammation in MIS-C. However, the long-term implications of cardiac sequelae in MIS-C are unknown but need a strict follow-up, as in KD [4].

At the follow-up, KD patients who developed CAL, show that aneurisms tend to regress, mostly medium-sized and small-sized aneurysms. The regression may be secondary to myointimal cells proliferation and/or to thrombus organization followed by recanalization. However, it is not possible to exclude long-term sequelae even in the non-complicated patients. Hence, it is strategic a long-term follow-up in KD patients with both persistent and regressed aneurysms with different timing and mode [5]. Children with KD-TSS have a higher incidence of CAL, than children without TSS. For these patients, the cardiologic follow-up is essential, also after the resolution of the acute critical phase.

The overlap of clinical features of MIS-C and KD induced researchers to consider the two conditions as the same disease, with the documented trigger of SARS-CoV-2 in MIS-C, and the suspicion of superantigens as the starting step in KD [6]. However, with the support of following studies, MIS-C was differentiated from KD on the bases of epidemiological, clinical, biochemical, and topographic records.

In fact, many children show respiratory symptoms or have an acute abdominal presentation. Intravenous immunoglobulin (IVIG) is recommended as first line therapy, as in KD. Recent evidence suggests IVIG resistance in some cases of MIS-C, thereby questioning the benefit of steroids and immunomodulators such as interleukin (IL) IL-1 or IL-6 blocking agents. Furthermore, the efficacy of anakinra in patients with a severe outcome of MIS-C, has a common pathogenetic underground with the results in non-responders or at high-risk patients with KD [7–9].

## Methods

We describe a cohort of 22 Sicilian children (11 M; 11 F; age: 1.4–14 years), with MIS-C, and a documented recent or actual infection by SARS-CoV-2, hospitalized from April 2020 to July 2021. The follow-up was performed for 10 months after discharge. Some cases showed shock and/or MAS secondary to a cytokine storm, fulfilling the CDC and WHO case definitions of MIS-C [1], including any of the following:

o positive SARS-CoV-2 RT-PCR
p positive serology
q positive antigen test
r contact with an individual with COVID-19.

Demographic and clinical data, laboratory, echocardiographic and imaging findings, treatment strategy and outcome were collected.

Common presenting symptoms included:

-fever, an essential criterion in the definition of MIS-C;
-abdominal pain and/or vomiting and/or diarrhoea, mucocutaneous rash, conjunctivitis, latero-cervical lymphadenitis, cheilitis and/or pharyngeal hyperaemia, hands and feet oedema.

Nasopharyngeal swab for SARS-CoV-2 was performed in all the patients and in their caregivers, and serological IgM and IgG-type antibodies against SARS-CoV-2 were dosed in all the children. Anamnestic records about positive parents’ swab were collected.

For this study, ethical review and approval was not required, because the data were retrospectively gathered.

### Statistics

 Patients were classified by gender and age; symptoms, clinical signs, drugs employed, treatment interval, were correlated with organ damage, ultrasound findings and normal or altered haematological parameters. Correlation between the analysed parameters collected were performed, using Chi-square test. All variables were tested for normal distribution, using the Anderson–Darling normality test. All ordinal data were expressed as numbers and percentages. Statistical analysis was done by “MiniTAB release 13.1 Statistical Software”.

## Results

Symptoms started 1–8 days before the hospitalization. The following diagnostic criteria were detected in the patients included in the study:

-fever (was present in 100% of the cases)
- cheilitis and/or pharyngeal hyperaemia (86%)
- latero-cervical lymphadenitis (82%)
- skin rash (73%)
- conjunctivitis (64%)
- abdominal pain and/or vomiting and/or diarrhoea (64%)
- hands and feet oedema (18%) (Table 1).

**Table 1:** Clinical signs and symptoms of the patients

Patients (age in years)	fever (days)	Rash	Conjunctivitis	lymphadenitis	cheilitis and/or pharyngeal hyperaemia	hands and feet oedema	abdominal pain and/or vomiting and/or diarrhoea	Arrhythmia	Pleuritis	Ascitis	Mesenteric adenitis	Pericarditis	Valvular insuff	CAL	MAS	Shock
1 (12.7) M	5	+	-	+	+ / +	-	-/-/-	+	-	+	+	-	+ (M)	-		
2 (6) F	6	-	+	+	-/ +	-	± /-	+	-	-	-	-	+ (M)	-		
3 (3) F	3	+	+	+	+ / +	-	-/-/-	-	-	-	+	-	-	-		
4 (2.8) F	3	+	-	+	+ / +	-	-/-/-	-	-	+	-	-	-	-	MAS	
5 (11) M	11.9	+	+	+	+ / +	-	** + / + / + **	-	-	-	-	-	-	+	-	-
6 (11) M	6	+	+	+	+ / +	-	+ / + / +	+	-	+	** + **	-	+ (T)	+		
7 (9) M	5	+	+	+	-/-	-	-/-/-	-	-	+	+	+	+ (M)	+		
8 (9.4) M	7	-	+	-	+ / +	-	+ / + / +	+	+	-	+	-	+ (M)	-		
9 (5.4) F	6	+	+	+	+ / +	-	+ / + / +	-	-	+	+	+	+ (M)	-	MAS	
10 (0.4) F	10	+	-	+	+ / +	+	+ / ±	-	+	-	-	-	-	-		
11 (1) M	7	+	+	-	+ / +	-	-/-/-	-	-	-	-	-	-	-		
12 (11.7) F	6	+	-	+	-/-	-	+ / + / +	+	-	+	+	-	+ (M)	-		
13 (2) M	6	+	+	+	-/ +	-	-/ ±	-	-	-	-	-	+ (A; M)	-		
14 (1.1) F	9	+	+	+	+ / +	+	-/-/-	+	-	-	-	-	-	-		
15 (14) F	4	+	-	+	-/ +	-	-/ + / +	-	-	-	-	-	+ (A; M)	+		
16 (11) M	12	-	-	+	-/ +	-	-/-/ +	-	-	-		+	+ (M)	-		
17 (3) F	5	+	+	-	+ / +	-	-/ ±	+	-	-	+	-	-	-		
18 (10.4) F	7	+	+	+	+ / +	-	+ / + / +	-	-	-	-	+	+ (M; T)	-		
19 (7.6) M	8	-	-	+	-/ +	+	± /-	-	+	+	-	-	-	-	MAS	Shock
20 (3.2) M	5	+	+	+	+ / +	+	-/-/-	-	-	-	-	-	-	-		
21 (14) F	6	-	+	-	-/-	-	-/ + / +	+	+	-	-	+	-	-	MAS	Shock
22 (1.5) M	4	-	-	+	+ / +	-	-/-/-	-	-	-	-	-	-	-		

Nasopharyngeal swab for SARS-CoV-2 was positive in 15/22 patients, who showed positive serological IgG, negative or grey zone IgM-type antibodies. 5 patients with negative swab had a history of recent infection and positive IgG-type antibodies; 2 patients had parents with positive swab, with no positive serological test, however fulfilling the CDC and WHO case definitions of multisystem inflammatory syndrome in children [1].

Infections other than SARS-CoV-2 were excluded in all the patients, by haemoculture and sept fast.

At the admission, a significant increase of C-reactive protein (CRP) and hyponatraemia was found in 100% of cases. AST, ALT, gamma-GT were above the range in 59% of the children. Increased pancreatic amylase and lipase levels were found in 14%, a lymphocyte count ≤ 1000 was documented in 36% of the patients enrolled.

Pro-BNP levels were increased (> 320 pg/ml) in 50% and troponin levels were increased (> 14 ng/ml) in 18% of cases.

D-Dimer was increased in all the patients, with a range of 0.56–13.36 mg/L (n.v. < 0.5); ferritin was increased (with levels > 365 ng/ml) in 11/22 (50%) of the patients.

IL-6 levels were evaluated in 14/22 patients and 13/14 showed a significant increase of IL-6 levels (30.2–285 pg/ml).

Furthermore, a mild kidney involvement was documented in 36% of the patients, with low-grade proteinuria in 8 and microhaematuria in 3 (Table 2).

**Table 2:** Laboratory parameters of the patients

Patients	Nasopharingeal Swab	IgM/IgG anti-SARS-CoV-2	Hb g/dl	WBC/µL	Neutrophils/lymphocytes/ µL	Platelets/µL	CRP (n.v. < 0.5 mg/dl)	Procalcitonin (µg/l)	Protein/albumin (g/dl)	Creatinine (mg/dl)	Na /K/ Cl (mmol/l)	AST /ALT (UI/l)
1	+	IgG +	13.9	8860	5883/1940	209,000	10.89	0,4	7.3/4.5	0.66	134/3.95/101	32/33
2	+	IgG + /I gM +	10.3	19,490	16,118/2163	157,000	23.78	7,39	6.2/3.5	0.43	132/4.21/100	22/9
3	+	IgG + /I gM +	10.3	9670	6720/2592	221,000	8.65	1,22	6.2/3.3	0.29	134/3.75/99	27/14
4	+		12.6	1910	850/1000	162,000	9.13	10.1	6.7/3.9	0.3	132/4.94/92	72/166
5	-	IgG + /I gM +		24,430	21,498/977	407,000	16.4		6.9/2.9	0.76	133/3.4/98	34/36
6	+	IgG + /I gM +	12.3	6230	5794/318	121,000	11.03	6.86	6,6/3.5	0.61	130/4.12/95	37/39
7	-	IgG + /I gM-	10.1	1129	857/151	138,000	10.58	17.27	6.2/2.8	0.45	128/3.79/99	51/30
8	+	IgG + /I gM +	12	4680	3800/580	70,000	12.03	5,91	6.8/3.7	0.53	127/3.65/90	24/57
9	+	IgG + /I gM ±	10	2090	1269/650	59,000	4.56	1.28	5.1/3	0.38	135/3.86/104	26/19
10	+		11.5	17,030	1450/13980	492,000	2.5		6.3/4.4	0.26	133/5.31/102	31/35
11	+	IgG + /I gM +	11.7	9650	3420/5170	224,000	5.76		5/3.3	0.53	132/4.6/99	60/22
12	-	IgG + /I gM +	14.7	8210	5120/2580	180,000	26.65		7.8/3.8	0.54	133/4.8/96	39/73
13	+	IgG + /I gM-	11.1	20,820	14,803/2311	293,000	3.56	3.37	7.2/3.7	0.4	132/4.4/100	105/53
14	-	IgG -/IgM -	12.5	5300	779/4182	169,000	1.88	0.57	6,1/3.7	0.28	132/4.01/102.4	63/64
15	+	IgG + /I gM +	12.3	10,340	2833/5201	309,000	0.29	< 0.05	7.9/4.3	0.77	133/2.25/99.3	18/21
16	+	IgG + /I gM-	11.2	16,860	14,769/1332	332,100	25.99	3.69	6.1/3.5	0.46	124/3.43/87	42/52
17	-	IgG + /I gM-	10.8	10,370	7715/1742	237,000	9.03		5.9/3.6	0.4	134/4.1/103	57/12
18	-	IgG + /I gM-	11.3	7470	6499/829	220,300	42.24		5.7/3.5	0.5	133/3.7/98	19/17
19	+		10.8	15,780	13,381/1594	210,000	27.2	0.31	6.9/3.5	0.53	124/4.22/86	273/254
20	+		10.6	7010	3870/2208	396,000	9.43	1.35	8.8/3.7	0.3	134/3.7/96	16/7
21	+		12.9	5710	3909/622	132,000	21.16	5.2	7/4.1	1.29	131/3.03/103	156/80
22	+		9	29,610	19,661/7284	394,000	17.29	0.66	8,1/4.4	0.34	133/5.57/97	25/9

Patients	CPK /LDH (UI/l)	Gamma GT (UI/l)	pancreatic amylase/lipase (U/L)	Troponine (ng/l)	PRO-BNP (pg/ml)	D- Dimer	Fibrinogen (mg/dl)	Ferritin (ng/ml)	Triglyceride (mg/dl)	IL-6 (pg/ml)	haematuria	Proteinuria
1	46/174	14	16/13	66.6	3908	1.49	560	386.8	279	16.7	-	-
2	61/348	11		5.8		2.112	645	187		285	+	+
3	8/382	12	33/39	3.39	1565		584	118.3	219	57.1	-	-
4	64/541	96	11/18	4.27	2193	1.79	525	364.4	745	65.6	-	+
5	34/331	46	98/38	3		2.13	151	1300	366		-	-
6	50/332	14	14/13	3		6.84	528	338.5	162		-	-
7	53/413	16	12/12.9	4.9	1559	5.5	550.6	659.1	77	233	-	-
8	334	17		8.58		13.36	527	1013	194	244	-	+
9	327	50	63/51	5.5	323	1.85	466	812	202	209	-	+
10	113/305	10	23/34			0.56	159				-	-
11			25/21.5			6	310	2357		280	-	-
12	21/282	34	269/278.7	8.64	2704	4.37	271	934	364	298	-	-
13	111/369	7		5.3	587	5.2	4497	124.1		649	-	+
14	502	21	23/34	6.01	129	2.73	368	135.6	117	30.2	-	-
15	136	17	11/18	3.3	67	1.139	342	10		2.82	-	-
16	14/357	96		409	726	1.91		696	180		-	+
17	95/301	10	69/51.9	7.35	930	1.12		147.6	117	76.1	-	-
18	65/268	19	38/33.2	29.7	3204	2.73		614.3		204	+	+
19	454/24	307	136	27.3		2.798		1445	212		-	-
20	46/164	7		3							-	-
21	12,542/445	51	32	12.5		2.86		815.7			+	+
22	31/413	10	6/11	< 3	702	2.272		104.4	70		-	-

Patients were divided in two groups: group A (with cardiac involvement: pericarditis, valvular damage, CAL) and group B (without cardiac involvement) (Table 3).

**Table 3:** M ± DS and significant *p*-value (in red) of haematochemical parameters, dividing the patients in two groups: group A (with cardiac involvement: pericarditis, valvular damage, CAL) and group B (without cardiac involvement)

Parameters	Value (M ± DS) Group A	Value (M ± DS) Group B	*p*-value
Hbg/dl	11.8 ± 1.5	11 ± 1.2	0.538
WBC/µL	10,486 ± 7486	11,814 ± 8175	0.698
Neu/µL	7934 ± 6566	6427 ± 6409	0.599
Lymph/µL	1512 ± 1368	4417 ± 4113	**0.027**
Plt/µL	202,100 ± 106,057	278,333 ± 117,782	0.129
CRP (n.v. < 0.5 mg/dl)	16.09 ± 11.61	7.1 ± 10.9	0.194
Procalcitonin (µg/l)	5.14 ± 4.99	2.37 ± 3.81	0.176
Protein (g/dl)	6.68 ± 0.81	6.67 ± 1.16	0.981
Albumin (g/dl)	3.6 ± 0.51	3.76 ± 0.41	0.444
Creatinine (mg/dl)	0.6 ± 0.24	0.36 ± 0.11	**0.011**
Na (mmol/l)	131 ± 3	132 ± 3	0.538
AST (UI/l)	47 ± 40	69 ± 79	0.381
ALT (UI/l)	40 ± 22	65 ± 87	0.331
Gamma-GT (UI/l)	55 ± 87	59 ± 104	0.913
Pancreatic amylase (U/l)	61 ± 83	39 ± 44	0.462
IL-6 (pg/ml)	230.35 ± 178.34	101.80 ± 101.06	0.066
LDH (UI/l)	315 ± 90	383 ± 123	0.155
Ferritin (ng/ml)	601 ± 418	667 ± 887	0.816
Fibrinogen (mg/dl)	804 ± 1248	530 ± 270	0.527
D-dimer (mg/l)	3.72 ± 3.37	2.47 ± 1.76	0.322

Proteinuria and microhaematuria were more frequent in patients of group A (7/13) than in of group B (1/9) (p-value: 0.0019). Furthermore, in group A lymphocyte count was lower (p-value: 0.027) and creatinine was higher (p-value: 0.0113).

Patients of group A had higher CRP levels (16.09 ± 11.61) than in group B (7.1 ± 10.9) and procalcitonin concentrations (5.14 ± 4.99) than group B (2.37 ± 3.81), even not reaching the statistical significance.

The cardiac involvement was documented by echocardiogram in 59% of children (pericardial effusion in 5; Mitral or Tricuspid insufficiency in 8; Mitral and Aortic insufficiency in 2; Mitral and Tricuspid insufficiency in 1; 4 with coronary artery lesions (CAL)). The Z score value was 3 in 1 patient; 3 patients showed a Z score < 3, with persistent brightness of the coronary wall. 4/5 (80%) of patients with pericarditis showed Mitral valve insufficiency as well. All the patients showed normal left ventricular ejection fraction. The ejection fraction of patients with cardiac involvement was > 60%. The 2 patients with shock did not show reduced cardiac contractility.

De-novo arrhythmia was documented in 8/22 children (36%): among those, tachycardia followed by persistent bradycardia was documented in 5 (23%), lasting for 8–10 days after the resolution of the acute phase of MIS-C. It was not correlated with increased levels of troponin and pro-BNP. Among those children who developed bradycardia, 2 showed alterations of the repolarization phase. Further 3 children showed alterations of the repolarization terminal phase or of the QRS, not known before. One patient showed a pre-existing Brugada pattern, revealed by fever with arrythmia.

Pleural effusion, ascitic effusion and mesenteric adenitis were found in 18%, 27% and 36% of the patients, respectively.

18% of patients dramatically and rapidly evolved in a MAS-like form, fulfilling the classification criteria for the diagnosis of MAS (ACR/EULAR 2016). High doses of steroids were promptly started and associated with IVIG, obtaining a significant improvement of the clinical course.

In all the patients, treatment was started within 72 h after the admission, with IVIG (2 g/Kg/dose), methylprednisolone (2 mg/Kg/day in 73% of patients; 30 mg/Kg/day for 3 days, followed by 2 mg/Kg/day in 27% of patients for the severe and acute progression of the MIS-C), acetylsalicylic acid (ASA) (3–5 mg/Kg/day). Two patients were treated with enoxaparin. Two patients with shock, were additionally treated with vasoactive drugs, albumin, diuretics. All the patients were treated with methylprednisolone at 1–2 mg/Kg/day until the normalization of inflammatory markers (CRP, ferritin, IL-6), troponin, D-Dimer, AST, ALT, gamma-GT, pancreatic amylase, lipase, lymphocyte count.

Hence, a gradual and slow tapering of methylprednisolone was performed, with the end of the therapy in 2–3 weeks. Treatment with low dose of ASA was maintained for 6–8 weeks, in patients with the resolution of CAL. The child with persistent CAL is still treated with ASA.

In this case series, no patients needed treatment with anakinra, indicated in patients who are refractory to IVIG and steroids, due to its efficacy in treating systemic inflammatory diseases.

All the patients were included in a follow-up, to investigate on clinical outcome and resolution of organ involvement. The follow-up was performed for a median time of 10 months and included medical examination, blood samples, ECG and echocardiogram, abdominal US.

Haematological parameters normalized in all the patients. The patients with increased pancreatic amylase showed the restore of pancreatic enzymes in 2 weeks. Proteinuria and/or haematuria resolved in 5 ± 2 days after the start of IVIG and steroids. Nobody showed a relapse of signs of kidney damage during the follow-up.

IL-6 levels showed a prompt normalization after steroids and IVIG treatment. At the contrary, the increase of pro-BNP levels persisted for 7–10 days after IVG and steroids treatment.

In all the patients, during the follow-up, we did not observe an inflammation rebound, with maintained normal levels of CRP, ESR, IL-6, ferritin, LDH, fibrinogen, D-Dimer. Troponin and pro-BNP levels, after the normalization, persisted in the normal range for all the period of the follow-up, as the other biochemical markers, also in patients with persistent valvular insufficiency or CAL.

Cardiac damage evolved into the complete resolution of lesions in most of the patients. The resolution was observed and documented by echocardiogram after 7–14 days since IVIG and steroids treatment was started. The time occurred between the diagnosis and the resolution of cardiac involvement was not correlated with biochemical parameters. One patient with pre-existing Mitral valve insufficiency had a persisting defect; in 1 patient, Mitral and Tricuspid insufficiency persisted over all the time of follow-up; 1 patient with pre-existing aortic insufficiency, worsened during the acute phase of MIS-C, showed the reduction of aortic insufficiency during the follow-up, while Mitral insufficiency reached the complete resolution. In 3 patients the Mitral insufficiency reduced but did not resolve during the follow-up.

In conclusion, 18% of patients with acquired valvular insufficiency persisted with the valvular impairment during the follow-up.

CAL resolved in 2 patients, within 2 weeks since IVIG and methylprednisolone treatment was started, persisted in 1, which is still treated with ASA at 5 mg/Kg/day.

The treatment strategy, the timing and the dosage of methylprednisolone did not differ between patients with and those without resolution of valvular or coronary lesions.

## Discussion

MIS-C is considered as a post-infectious inflammatory response following SARS-CoV-2 infection. It shows a different clinical presentation, linked to cytokine storm occurring weeks later the contact with the virus. The patients with MIS-C usually do not have anamnestic records of co-morbidities and have a common clinical presentation, associated with gastrointestinal symptoms, as abdominal pain, vomiting and/or diarrhoea. The cardiac involvement can vary, between cases with CAL, cases with valvulitis, pericarditis and/or shock.

The clinical presentation is widely different from severe acute COVID-19 in paediatric patients, who are younger, can manifest comorbidities, upper and low airway symptoms and respiratory dysfunction [10].

These patients show a wide range of presenting symptoms and signs. They show the evidence of inflammation with increased values of erythrocyte sedimentation rate (ESR), CRP, IL-6, ferritin, D-dimer, pro-BNP, troponin, transaminase, pancreatic amylase and albumin. A multi-organ involvement was described in most of cases, in some cases with an overlap with acute SARS-CoV-2 infection, requiring a multi-specialistic approach [11]. Furthermore, the mild kidney involvement was reported as an organ involvement in children with MIS-C [12]. In our patients it resolved in a few days after the immunomodulatory treatment.

Resolving systemic inflammation, pro-BNP usually improves within a few days, troponin may persist high for longer until the regenerative repair of apoptotic cells is completed. Generally, pro-BNP and troponin levels in MIS-C are higher than in KD and reflect the extent of vasculopathy and cardiomyocytes damage [13]. Persistence of increased levels of pro-BNP, in patients with a normalization of inflammatory parameters, suggests a mechanism of myocardial oedema, persisting besides the intensive care approach, able however to limit effects on cardiac function and normalize inflammatory parameters [14].

An open question is about the long-term cardiac complications in children with MIS-C. Children treated with immunomodulators showed a favourable outcome in most cases, with recovery of pericardial effusion, CAL, valvular insufficiency, left ventricular contractility [13–15]. We still not know if the cardiac lesions observed in the acute phase of the disease are persistent or transient, and if they are the beginning of a persistent cardiomyopathy.

During the 10 months follow-up we documented a persistence of valvular impairment in 18% of patients in with valvular inflammation was documented during the acute phase of MIS-C.

The international literature documented that the early evidence of pericarditis in KD is significantly correlated with CAL onset [16]. Thereby, pericarditis must be considered an alarm and precocious sign of CAL and/or valvular damage in children with KD. In our patients with MIS-C, 80% of patients with pericarditis showed contemporary mitral valve insufficiency. This discrepancy between the two conditions, commonly considered as “twin diseases” for the clinical presentation, the laboratory findings and the treatment choices, highlights intrinsic differences between KD and MIS-C. In fact, MIS-C typically shows an acute and severe cardiac involvement in most critical cases. Conversely, CAL can develop also during the follow-up in KD. Echocardiogram in the first week of illness is typically normal and does not exclude KD diagnosis. In fact, KD vasculitis is caused by diffuse neutrophil infiltration with necrotizing features of small arteries, which might potentially lead to vessel dilatation generally occurring during the subacute phase [17].

In our patients, cardiac valve involvement persisted only in 18% of children, CAL persisted only in 33% of children with coronary involvement, however without the evolution into aneurisms. In our Sicilian children with KD, we documented a higher incidence of persisting CAL [16], suggesting that cardiac involvement in MIS-C manifests at the beginning and seems more severe in the acute phase of the disease, while tends to resolve in a higher percentage of children. These data need to be confirmed in a longer period of follow-up. However, the persistence of valvular heart disease proves that post-inflammatory fibrosis can maintain the endothelial damage in heart tissues. The treatment strategy and timing did not differ between patients with resolution and those with persisting injuries.

It is a key matter to investigate the factors associated with the resolution of CAL, valvular damage, and myocardial dysfunction in children with MIS-C. The drugs employed and the timing of therapeutic approaches can contribute to resolve the cardiac lesions. The cornerstone for an effective treatment of MIS-C could be the early use of a good immunomodulatory therapy. An open question is if there is a “golden hour” for starting the treatment [18].

The preferred treatment strategy was more aggressive at the diagnosis of MIS-C, to block the cytokine cascade. Most of our patients, in fact, received a first-line treatment with IVIG and steroids. This approach could explain the favourable prognosis, the rapid restoring of cardiac function also in patients with MAS or shock, and the good outcome during the 10 months follow-up in all the patients.

All the patients did not show persistent organ damage nor inflammatory rebound during the follow-up, sustaining the benefits of the treatment strategy adopted in these cases.

Treatment with methylprednisolone at 30 mg/kg/day, was chosen in 27% of our patients, for the severe and acute presentation of the MIS-C. We strictly followed the patients and obtained a good response to treatment. For this reason, anakinra was not indicated, also for the favourable course of the disease.

## Conclusions

Further studies on long-term outcomes of those patients are needed to understand the disease and to perform definitive guidelines for the management of MIS-C.

More studies are necessary to evaluate if persistent or transient cardiac complications are associated with long-term adverse cardiovascular events in adulthood, and if genetic imprinting can influence the outcome.

## Data Availability

The datasets used and/or analysed during the current study are available from the corresponding author on reasonable request.

## Abbreviations

MIS-C: Multisystem inflammatory syndrome in children
CAL: Coronary artery lesions
IVIG: Intravenous immunoglobulins
ASA: Acetyl salicylic acid
SARS-CoV-2: Severe acute respiratory syndrome coronavirus 2
KD: Kawasaki disease
TSS: KD-related shock syndrome or toxic shock syndrome
MAS: Macrophage activation syndrome
CRP: C-reactive protein
ESR: Erythrocyte sedimentation rate
IL: Interleukin
